# Perspectives of GCSE students attending a psychiatry summer school in south London

**DOI:** 10.1192/bjb.2020.76

**Published:** 2021-04

**Authors:** Clementine Wyke, Glori-Louise de Bernier, Chun Chiang Sin Fai Lam, Clare Holt, Sophie Butler, Anto Praveen Rajkumar Rajamani, Charlotte Wilson Jones

**Affiliations:** 1 South London and Maudsley NHS Foundation Trust, UK; 2 University of Nottingham, UK; 3 Institute of Psychiatry, Psychology and Neuroscience, UK

**Keywords:** Education and training, recruitment, summer school, stigma and discrimination, school students

## Abstract

**Aims and Method:**

This study evaluated a pilot psychiatry summer school for GCSE students in terms of participant experience, effects on attitudes to mental illness and perception of psychiatry as a career option. This was done using the Community Attitudes towards the Mentally Ill scale, career choice questionnaires and a discussion group following the week-long programme attended by 26 students.

**Results:**

Students were significantly more likely to choose psychiatry after the summer school (*P* = 0.01). There were statistically significant changes in scores for social restrictiveness (*P* = 0.04) and community mental health ideology (*P* = 0.02). Qualitative analysis generated four themes: variation in expectations, limited prior knowledge, perception of the summer school itself and uniformly positive attitudes to psychiatry after the summer school.

**Clinical implications:**

Targeting students at this early stage appears to be an underexplored positive intervention for improving both attitudes towards mental illness and recruitment to psychiatry.

## Introduction

Summer schools allow students to access educational experiences that are not otherwise available to them. They are a successful facet of the Royal College of Psychiatrists’ #ChoosePsychiatry campaign^1^ and, if well designed, can improve attitudes to psychiatry among medical students.^2^ However, as summer schools are primarily aimed at medical students and foundation doctors, opportunities to experience psychiatry while at school remain limited. A psychiatry scheme for school leavers^3^ and work experience placements focusing on mental health for A-level students^4^ have been reported with positive outcomes. However, these examples concentrate mostly on those already committed to applying to medical school. Such programmes, especially those with entrance processes requiring evidence of prior interest, tend to favour those already motivated to work in psychiatry. Thus, there is recruitment potential in prioritising those that have little prior knowledge of the subject.^5^ Studies have shown that medical students reporting an interest in psychiatry before medical school are more likely to subspecialise in it in the future.^6,7^ Therefore, the evidence suggests that a summer school for students following their GCSEs would allow them to obtain valuable early experience of psychiatry and hopefully garner a potential untapped interest in psychiatry as a career. The aim of this study was to evaluate the experiences of GCSE students attending a psychiatry summer school, with a secondary aim of assessing its influence on attitudes to mental illness and perception of psychiatry as a career.

## Methods

### Setting

The summer school was a joint venture by the Institute of Psychiatry, Psychology and Neuroscience (IoPPN) and the local mental health trust, South London and the Maudsley NHS Foundation Trust. It was a free 5 day programme (see Table 1) for GCSE students, with participants recruited from state and private secondary schools within south-east London.

**Table 1:** Summer school timetable

Monday	Tuesday	Wednesday	Thursday	Friday
Introduction to Mental Health 0930–1030 Welcome session Break 1045–1215 Breakaway groups on ‘What is Illness?’ and ‘What is mental illness’?	Psychiatry & Society 0900–1000 The evolution of consciousness 1015–1115 History of Psychiatry Break 1130–1245 Psychiatry Case 2 (forensic patient)	Life as a Psychiatrist 0915–1030 From start to finish *Psychiatrists at varying stages of their careers* Break 1100–1230 Psychiatry Case 3 (patient with FND)	Psychiatry Specialties 0900–1100 Quick Fire Specialties *Psychiatrists working in subspecialties, including psychotherapy, affective and psychotic disorders, addictions and eating disorders* Break 1115–1230 Psychiatry Case 4 (CAMHS patient)	Looking to the Future 0900–0930 Old Age Psychiatry 0930–1000 Thoughts on Psychiatry 1000–1115 Psychiatry Case 5 (patient with psychosis) Break 1130–1300 Debate (joint with medical students) ‘This House believes that social media damages your mental health’
LUNCH	LUNCH	LUNCH	LUNCH	LUNCH
1330–1430 What is Psychiatry? 1430–1500 Myth Busting Break 1515–1630 Psychiatry Case 1 (patient with PTSD) *Consultant psychiatrist & their patient*	1400–1430 Global Mental Health 1430–1715 Psychiatry in the Arts	1345–1600 Extreme Psychiatry (joint with medical students)	1330–1430 Psychiatry of homelessness 1430–1530 Military Psychiatry Break 1600–1700 Neuropsychiatry	1400–1600 Life at Medical School & How to Get There *Medical school psychiatry society* 1600–1630 Feedback and discussion group

CAMHS, child and adolescent mental health services; FND, functional neurological disorder; PTSD, post-traumatic stress disorder.

### Selection of participants

Letters were sent to careers advisors at all 15 eligible schools within a 1.5 mile radius of the IoPPN, inviting them to nominate two participants and two waiting-list students each for the summer school. Non-eligible schools were those that only catered for pupils with special education needs or were sixth form only. Each school independently decided on the selection procedure, with the only proviso being that the student should be academically capable of entering a medicine degree course. They did not have to have expressed any interest in medicine as a career.

### Curriculum development and structure of summer school

The curriculum was developed and implemented by a volunteer committee of psychiatry trainees, led by the Director of Undergraduate Psychiatry. The programme included a mixture of lectures and small group workshops, in addition to three joint sessions with an established summer school for medical students being held in parallel. Content included daily sessions with patients and their psychiatrists and the breadth of psychiatry subspecialties, but also addressed the wider context of mental illness such as social factors, transcultural applications and the history of psychiatric practice.

### Assessment

This was a mixed quantitative and qualitative methods evaluation with ethical approval obtained through the Research Ethics Office at King's College London.

All participants were invited to take part in the evaluation, and participants gave written informed consent. Pre-programme, demographic information and prior exposure to mental illness were collected. Participants’ top three career choices, likelihood of choosing a career in psychiatry and score on the Community Attitudes towards the Mentally Ill (CAMI) scale^8^ were obtained pre and post programme.

The CAMI scale comprises 40 items representing the following four dimensions: authoritarianism (the view that the mentally ill are different and require coercive measures), benevolence (sympathetic views towards the mentally ill), social restrictiveness (that the mentally ill are dangerous and need to be separated from society) and community mental health ideology (importance of community care for the mentally ill).^8,9^ It was selected for its utility within a community rather than a professional population, as the sample consisted of school students with no medical training.

On the final day, a short discussion group was run to gather feedback on the summer school experience. All students were informed of the group, and six randomly selected volunteers contributed. The facilitator (G.-L.d.B.) was known to the students, so left the room after posing each question to enable free unconstrained responses, which were recorded.

The contributors were asked three open questions, which were designed to enable discussion and aimed to assess ‘before and after’ attitudes in relation to the summer school and psychiatry.

• Why did you want to come to the summer school and are you glad that you came?
• What did you hope to get out of the summer school and what did you actually get out of it?
• How did you feel about psychiatry before and after the summer school?

### Statistical analyses

Only one of the participants did not complete post-training preferences for medicine and psychiatry, and these missing values were not imputed. Participants’ characteristics and other variables were initially analysed by descriptive statistics. We checked whether the continuous study variables followed a Gaussian distribution by Shapiro–Wilk tests. We employed appropriate non-parametric tests when the continuous study variables did not follow a Gaussian distribution. Changes in the career choices of the participants between the two time points were analysed by McNemar's test or Wilcoxon signed-rank test. Changes in CAMI subscale scores between the two time points were analysed by appropriate tests of statistical significance. All analyses were performed using the statistical software STATA 15.1 (StataCorp, TX, USA).

After transcription of the discussion group, the responses were initially analysed using open coding by a researcher independent of the summer school programme (C.H.). Alongside a second researcher, who had overseen the discussion (G.-L.d.B.), these open codes were grouped into axial codes, which were then further distilled into themes.

## Results

### Quantitative

Of the 26 participants, ten (38%) were from private schools and the remainder were from state schools. Of the participants, 20 (77%) voluntarily completed both the pre- and post-programme questionnaires. Of these students, 12 (60%) were from non-White ethnicities. Data on demographics and career choices can be found within Tables 2 and 3. The majority (70%) reported life experience of mental illness before attendance at the summer school, and 50% had received some school teaching on the subject.

**Table 2:** Demographic characteristics

Demographic characteristic	*n* (%)
Total number	20
Gender	
Male	7 (35)
Female	12 (60)
Not specified	1 (5)
Age (years)	
15	1 (5)
16	19 (95)
Ethnicity	
White	8 (40)
Mixed	2 (10)
Black/African/Caribbean	6 (30)
Asian	4 (20)
Experience in mental health	
Life experience (personal/friends/family)	14 (70)
Work experience/volunteering	2 (10)
School teaching on mental health	10 (50)
Family member working in mental health	6 (30)

**Table 3:** Career choices

Before programme (*N* = 20)			After programme (*N* = 19)		
Choice	*n*	%	95% CI^a^	*n*	%	95% CI^a^
First	2	10.00	1.23–31.70	5	26.32	9.15–51.20
Second	2	10.00	1.23–31.70	2	10.53	1.30–33.14
Third	0	0.00	0.00–16.84^b^	2	10.53	1.30–33.14
Within top 3	4	20.00	5.73–43.66	9	47.37	24.45–71.14

a. Binomial exact confidence interval.

b. One-sided 97.5% confidence interval.

According to the measure of a student's likelihood to choose psychiatry as a career, participants were significantly more likely to choose psychiatry after the summer school week (*z* = 2.46; *P* = 0.01). Pre-course, four participants ranked psychiatry within their top three career choices. This increased to nine post-course, which was a statistically significant change (McNemar's χ^2^ = 5.00; *P* = 0.03). Two participants ranked psychiatry as their top career choice pre-course, and this increased to five post-course. However, this change was not statistically significant (McNemar's χ^2^ = 3.00; *P* = 0.08).

On review of the CAMI scale, there were statistically significant changes in scores pre- and post-programme for both social restrictiveness (viewed less positively, pre: 18.6, post: 16.05, *t* = −2.25; d.f. = 19; *P* = 0.04) and community mental health ideology (viewed more positively, pre: 38.45, post: 40.5, *t* = 2.48; d.f. = 19; *P* = 0.02). There were no significant changes for benevolence (pre: 41.15, post 41.55, *P* = 0.54) or authoritarianism (pre: 20.8, post: 19.8, *P* = 0.33).

### Qualitative

Qualitative analysis of the discussion group generated 29 open codes and ten axial codes, from which four themes were generated.

#### Theme 1: Variation in expectations of summer school

The group was divided between those who had positive expectations for the programme and others who described initially feeling less enthusiastic about attendance. Those with an existing interest in medicine or mental health tended to express expectations that the summer school would enhance their knowledge of mental health conditions and provide further insight.

‘*I definitely wanted to go into medicine so I just wanted to see about the different areas’*

‘*I hoped to get more informed about different mental disorders…because I've been interested in that for a while*’

Others had a more generic reason for signing up to the programme, with half mentioning wanting to keep themselves occupied over the summer holiday or participate in an activity that was both enjoyable and worthwhile. One participant admitted being coerced by his mother to attend. The students discussed their negative preconceptions; some anticipated that the sessions would be wholly didactic in nature, with senior doctors leading and no element of interaction. There was also a concern voiced that, as school students, they may have felt patronised.

‘*My expectation coming in was that it would be a lot less involved than I thought it would be. I thought it would just be doctors talking over us instead and to us instead of actually letting us discuss’*

‘*I hoped that I would be treated in a way that wasn't a GCSE student that's like dumb and doesn't know anything, doesn't really know what they want to do and over dumbed-down for them’*

#### Theme 2: Limited prior knowledge and exposure to psychiatry

As a group, the participants admitted to very limited previous knowledge about mental illnesses, the scope of psychiatry and the management options available. Most brought up a lack of understanding of the difference between the disciplines of psychology and psychiatry, including those who had been exposed to the topic in lessons and school talks.

‘*I didn't know that a psychiatrist was actually a doctor, I didn't know that mental illnesses were such a wide range and they were so important and there are different ways of dealing with them’*

In their personal lives, there was one suggestion of first-hand experience of mental health difficulties and one student whose parent worked in the field. However, familiarity was not necessarily advantageous – the aforementioned parent allegedly refused to talk about their job to their child. Some referenced impressions of psychiatry that had been created and influenced by the media.

‘*I thought it [psychiatry] was about medicating people and torturing them in a way and putting them to sleep (laughing) I'm serious, I'm actually being serious, because of the movies’*

#### Theme 3: Perception and experience of summer school

Perception of the summer school retrospectively was consistently positive across the cohort. All felt they had benefited in at least one respect, such as better knowledge of mental health conditions, increased interest in the area or even a sense of privilege from involvement in the programme.

Repeated comments were made about the rare opportunity to associate with professionals and medical students, which was found to be a valuable way of gaining insight from those directly involved in clinical work. Interest was expressed in not only the substance of their work but also personal experiences and perspectives.

‘*…to talk to actual patients, talk to actual doctors, actual medical students and I think it's a really amazing opportunity and I wish there were more that were just as easily accessible and just as free and as local’*

The daily sessions with past or current patients exploring their experiences of mental illness and treatment were frequently mentioned as a highlight of the programme. The participants appreciated the university-style teaching methods, particularly the interactive components and being given space for further discussion on the topics broached.

‘*…we have had the opportunity to talk and express our own opinions about other stuff which I didn't think we'd be able to, and while doing that, also shown a lot of really good stuff about what it's like to be a doctor or a psychiatrist’*

‘*We were in actual lecture theatres listening to lectures university style, we were looking at patients… It was really amazing, I got a lot more knowledge and experience out of this week then I could have possibly thought. I just hoped I'd get an opportunity to talk about psychiatry but nothing on this kind of scale’*

#### Theme 4: Uniformly positive attitudes towards psychiatry following summer school

In addition to their experience of the summer school as a programme, all participants had a positive impression of psychiatry in itself. Those who began with a limited or negative perspective indicated that prior misconceptions had been challenged and questions had been answered. Psychiatry was compared favourably with other areas of medicine, and psychiatrists as a group were looked upon positively.

‘*The treatment of the patients has been fulfilling to them [psychiatrists] personally as well. It might be a lot more interesting than the other professions or the other parts of being a doctor, which I guess is a change’*

‘*It's also the relationships that they have with the patients. They talk about it like they actually remember them and they actually care which is really nice to know’*

Although not specifically asked about career intentions in the discussion group, most volunteered that they were considering psychiatry as an option for the future as a result of the summer school. This included those who were not previously interested in medicine and also those who had considered becoming a doctor but were focused on other branches of practice. Some had even forged interests in specific subspecialties.

‘*From the very first day and the very first session, my eyes were really opened as to what exactly psychiatry is about and how it can appeal to somebody like me, especially considering I wanted to be doing a different kind of speciality, like surgery, but now I think I have a much more open viewpoint on the different specialities, especially psychiatry’*

‘*This week has changed my perspective so much that I'm actually wanting to go into military psychiatry so yeah it's changed me as a person’*

## Discussion

Our evaluation of this inaugural summer school has demonstrated that an educational project such as this has the potential to encourage students yet to start their A-levels to consider a career in psychiatry.

Despite increasing awareness of the importance of mental health in the public domain, participants showed limited knowledge about mental illnesses, including misconceptions about the role of a psychiatrist, a lack of knowledge about treatment options and influence from negative media portrayals of the profession. This indicates that campaigns and media interest^10^ do not necessarily translate into awareness of career opportunities, which must be a separate strand of work in parallel with stigma reduction. The improvement in understanding and awareness shown by the time of the discussion group demonstrates the direct effect of the summer school.

Although clinical contact has not always been an essential factor in improving attitudes towards psychiatry,^2^ our experience was that witnessing the doctor–patient relationship and hearing about the effects of the work of a psychiatrist were key strengths of the summer school identified by the participants. In keeping with medical student and trainee experience,^11^ it appears that role models are also important early on to enable students to visualise their own potential next steps.

This evaluation adds to the body of evidence that educational interventions can change the attitudes of adolescents towards mental illness.^5,11–15^ Although this was not the primary purpose of the summer school, it was a welcome side-effect. It is noted that not all subscales of the CAMI showed significant change pre and post course. However, given how little current comparative data exist on attitudes to mental illness in our population group, further research is required to fully explain these results.

The Royal College of Psychiatrists’ current recruitment strategy^1^ is inclusive of school students, having aimed to roll out regional sixth-form career events by the end of 2019. We propose that GCSE student events are an area worth exploring further. These may serve to inspire students who perhaps have a less fixed commitment to one career path, have not considered medicine previously or have little knowledge of the scope of psychiatry and still have time to change their A level choices. We felt the following aspects were crucial to making the summer school accessible to a range of students and meeting the national efforts to widen participation.^16^

• Enabling students of all socioeconomic backgrounds to participate; it was free of charge and food was provided.
• Ensuring there was no competition between private and state schools for places.
• Proactive chasing of schools that did not immediately respond (these tended to be state schools, who were less likely to have a dedicated careers advisor).
• Timing the summer school before A-level choices so students had the option to change these if they wanted.

The main limiting factor in this study was the small sample size of 20 students. Our qualitative data from the discussion group did not reach saturation, demonstrating that not all potential data were garnered from this aspect of the evaluation. More discussion groups would be required in further research. In addition, although our cohort comprised a mixed demographic from a diverse part of south-east London, even if they had a variety of motivations, expectations and career interests, the students were still a self-selecting group, with all agreeing to attend a week-long summer school on psychiatry. We did not collect information such as family history of higher education and parental occupations, but doing so would help to assess whether students from all socioeconomic backgrounds were accessing the summer school. A barrier to transferability is that areas without potential funding from a major educational institution such as the IoPPN and a large pool of academics and clinicians to draw upon may find it challenging to provide this intensity of programme for a relatively small number of participants. Finally, as this is a little-studied population, it is unclear which assessment tool is most suited to explore school students’ attitudes towards psychiatry.

## Conclusion

Targeting students at this early stage appears to be an under-utilised intervention for psychiatry recruitment, and one which offers exciting potential for further work. The participants reported universally positive experiences of the summer school and demonstrated a shift towards considering psychiatry as a future career. There was also a valuable side-effect of more positive attitudes towards those with mental illness. We plan to repeat this summer school in future years and undertake longer-term follow-up in regard to participants’ A-level and degree choices and ongoing interest in psychiatry as a career.

## About the authors

**Clementine Wyke** is a CT3 in Psychiatry at South London and Maudsley NHS Foundation Trust, Maudsley Hospital, London, UK. **Glori-Louise de Bernier** is a CT3 in Psychiatry at South London and Maudsley NHS Foundation Trust, Maudsley Hospital, London, UK. **Chun Chiang Sin Fai Lam** is a Consultant Liaison Psychiatrist at South London and Maudsley NHS Foundation Trust, King's College Hospital, London, UK. **Clare Holt** is a General Adult and Old Age Psychiatry Registrar at South London and Maudsley NHS Foundation Trust, Maudsley Hospital, London, UK. **Sophie Butler** is a General Adult Psychiatry Registrar, South London and Maudsley NHS Foundation Trust, Maudsley Hospital, London, UK. **Anto Praveen Rajkumar Rajamani** is a Clinical Associate Professor in Old Age Psychiatry at the Institute of Mental Health, University of Nottingham, Nottingham, UK. **Charlotte Wilson Jones** is Director of Undergraduate Psychiatry & Director of Mental Health Education (MBBS) at the Institute of Psychiatry, Psychology & Neuroscience: Academic Division, UK.
